# Expression of epithelial cell adhesion molecule and proliferating cell nuclear antigen in diethylnitrosamine-induced hepatocarcinogenesis in mice

**DOI:** 10.3892/etm.2012.751

**Published:** 2012-10-17

**Authors:** JIN SEOK KANG, HWAN-GOO KANG, YOUNG-IL PARK, HYUNJUNG LEE, KIHO PARK, YUN-SEOK LEE, SOOHEE KIM, DOUG-YOUNG RYU

**Affiliations:** 1 Department of Biomedical Laboratory Science, Namseoul University, Cheonan 330-707;; 2 Toxicology Laboratory, Toxicology and Residue Chemistry Division, Animal, Plant and Fisheries Quarantine and Inspection Agency, MIFAFF, Anyang 480-757;; 3 Laboratory of Genomics and Quantitative Real-Time PCR, Biomedical Research Institute, Seoul National University Hospital, Seoul 110-744;; 4 Department of Health Administration, Namseoul University, Cheonan 330-707;; 5 College of Veterinary Medicine, Seoul National University, Seoul 151-742, Republic of Korea

**Keywords:** mouse, hepatocarcinogenesis, epithelial cell adhesion molecule, proliferating cell nuclear antigen, *in vivo*, *in vitro*

## Abstract

To clarify the role of stem cells in hepatocarcinogenesis, the expression of epithelial cell adhesion molecule (EpCAM) and proliferating cell nuclear antigen (PCNA) was investigated in mouse hepatic tumors and embryonic cell lineages. Ten ICR mice were treated with diethylnitrosamine (DEN) at 14 days of age and sacrificed at 36 weeks subsequent to DEN treatment to obtain the hepatic tumors. Mouse embryonic stem cells, hepatic progenitor cells and hepatocyte-like cells, representing 0, 22 and 40 days of differentiation, respectively, were treated *in vitro* with DEN at four doses (0, 1, 5 and 15 mM; G1, G2, G3 and G4, respectively) for 24 h and RNA was isolated. A total of 71 hepatic tumors were obtained from the DEN-treated mice. EpCAM expression was increased mainly in hepatic tumor cells, although it was also detected in the surrounding visually normal cells. Double staining showed that EpCAM and PCNA were co-expressed in numerous tumor cells. *In vitro*, EpCAM expression was significantly different for G4 at day 0 (P<0.01) and for G2, G3 and G4 at day 40 (P<0.01) compared with the control (G1) at the corresponding time-point. PCNA expression was significantly different for G3 and G4 at day 0 (P<0.01), for G2, G3 and G4 at day 22 (P<0.01) and for G2 at day 40 (P<0.01) compared with G1 at the corresponding time-point. In summary, the expression of EpCAM and PCNA was increased in DEN-induced tumors and the expression of EpCAM and PCNA was altered by DEN treatment in cultured cells. This suggests that EpCAM expression may be modulated in the progeny of adult liver stem cells during their differentiation toward hepatocytes and may be increased during DEN-induced hepatocarcinogenesis.

## Introduction

Carcinogenesis is considered to be a multi-stage process involving the initiation, promotion and progression of tumor cells, which may arise as a result of DNA damage, mutations, clonal expansion of preneoplastic cells and dysregulation of oncogenes and/or tumor suppressor genes.

Embryonic stem cells (ESCs) have self-renewal potential and may be differentiated into lineage-specific cell types, including hepatocytes (1). In the liver, stem cells are located in the ductal plates in fetuses and neonates and in the canals of Hering in infants and adults (2). As stem cells have the potential to survive, DNA damage induced by carcinogens may remain as cell proliferation occurs. Furthermore, stem cells with a loss of DNA repair function may be susceptible to malignant transformation, either directly or through the emergence of cancer-prone stem cells (3). Cell proliferation at the time of carcinogen exposure may be pivotal for the fixation of genotoxic injury as a heritable form (4).

It has been proposed that hepatocellular and ductal carcinomas originate from liver stem cells and that enzyme-altered foci and nodular changes are adaptive non-oncogenic responses to the toxic effects of carcinogens (5). Liver tumors appear to be hierarchically organized and sustained by a distinct subpopulation of cancer stem cells (6). This suggests that stem cells have a significant role in hepatocarcinogenesis.

As a putative stem cell marker, the epithelial cell adhesion molecule (EpCAM) is a membrane glycoprotein highly expressed on the majority of cancer cells (7), although it is also expressed on the majority of normal epithelial cells. In humans, EpCAM-positive hepatocytes have been found to be rare in the early stages of liver disease. However, they became increasingly prominent during the later stages and are consistently arrayed around the periphery of cords of keratin 19-positive hepatobiliary cells in the ductular reaction (8). Human EpCAM-positive hepatocellular carcinomas (HCCs) exhibit a distinct molecular signature with the features of hepatic progenitor cells (HPCs), including the presence of known stem cell/progenitor cell markers, including cytokeratin 19 and c-Kit, whereas EpCAM-negative HCCs exhibit a gene expression profile with the features of mature hepatocytes (9). Thus, EpCAM expression may be elevated during human liver tumor progression.

EpCAM expression in mouse liver carcinogenesis has not yet been reported. In the present study, we investigated the expression of EpCAM in diethylnitrosamine (DEN)-induced hepatic tumors. DEN is widely used in mouse liver cancer models (10) and has been used in several mouse strains (11–14). Based on the finding that young animals treated with DEN exhibited a higher incidence of liver tumors than older animals (15), we also assessed the effects of DEN on the expression of EpCAM and proliferating cell nuclear antigen (PCNA) in hepatic cells. The assessment was carried out at various developmental stages, from ESCs to HPCs and hepatocyte-like cells (HCs).

## Materials and methods

### Animals and treatment

ICR mice (Koatec Inc., Pyungtaek, Korea) were housed in a room maintained on a 12 h light/dark cycle and at a constant temperature and humidity. The mice were allowed free access to a pellet chow diet (Koatec Inc.) during the experiment. Male mice were bred with females, yielding the F1 generation. Male F1 mice at 14 days of age were injected intraperitoneally with DEN (10 mg/kg body weight; Sigma, St. Louis, MO, USA) dissolved in 0.9% saline. Ten mice treated with DEN were sacrificed 36 weeks later and hepatic masses were sampled for histopathological examination.

### Histopathological examination of hepatic tumors

The hepatic masses (n=71) were fixed in 10% neutral phosphate-buffered formalin, embedded in paraffin, sectioned to a thickness of 4 *μ*m and stained with hematoxylin and eosin. Tumor characteristics were classified based on histopathological and cytological criteria.

### Immunohistochemical analysis of EpCAM in hepatic tumors

The avidin-biotin complex method was used to stain EpCAM in 4-*μ*m sections of liver tissues. The sections were dewaxed in xylene, hydrated using a graded ethanol series and boiled in sodium citrate buffer (pH 6.0) in an autoclave for 20 min. Then, they were sequentially treated with 0.3% hydrogen peroxide, blocking buffer containing skimmed milk and the anti-EpCAM antibody (ab32392; Abcam, Cambridge, MA, USA; diluted 1:400). The sections were washed with TBS-T and subjected to the ABC-peroxidase procedure (ABC kit; Vector Laboratories, Burlingame, CA, USA). As a negative control, skimmed milk was used instead of the primary antibody.

The immune complexes were visualized using the chromogen 3,3′-diaminobenzidine tetrahydrochloride (DAB). The sections were counterstained with hematoxylin to facilitate their examination under a light microscope.

### Double staining of EpCAM and PCNA in hepatic tumors

The sections were dewaxed in xylene, hydrated using a graded ethanol series and boiled in a sodium citrate buffer (pH 6.0) in an autoclave for 20 min. Then they were sequentially treated with 0.3% hydrogen peroxide, blocking buffer containing horse serum and anti-PCNA antibody (M879; Dako, Carpinteria, CA, USA; diluted 1:500) for 1 h. The sections were washed with TBS and incubated with HRP-polymer (MRT621; Biocare Medical, Concord, CA, USA). The immune complexes were visualized using DAB.

For concomitant labeling of EpCAM, the sections were washed with TBS, blocked with goat serum, incubated with anti-EpCAM antibody (ab32392, Abcam) for 1 h and then incubated with AP-polymer (RMR625; Biocare Medical) for 30 min. The sections were washed with TBS, treated with Vulcan Fast Red (FR805H; Biocare Medical), washed again with TBS, counterstained with hematoxylin and viewed under a light microscope.

### Culture of mouse ESCs and differentiation of hepatic lineage cells

Mouse NVRQS-11F ESCs were cultured on mitomycin C-treated mouse embryonic fibroblasts (feeder cells) grown on 0.1% gelatin-coated dishes in Dulbecco’s modified Eagle’s medium (Millipore, Billerica, MA, USA), supplemented with 15% fetal bovine serum (Hyclone, Rockville, MD, USA), 2 mM L-glutamine (Millipore), 0.1% non-essential amino acids (Invitrogen, Carlsbad, CA, USA), 1% penicillin-streptomycin (Millipore) and 10 ng/ml mouse leukemia inhibitory factor (Millipore).

To differentiate the ESCs into hepatic lineage cells, we used the culture conditions for differentiating ESCs into HPCs and HCs reported by Zhou *et al*(16).

### DEN treatment of ESCs, HPCs and HCs

To determine a non-cytotoxic concentration of DEN, ESCs were treated with DEN at concentrations of 0–90 mM for 24 h. Cell viability was estimated by 3-(4,5-dimethylthiazol-2-yl)-2,5-diphenyltetrazolium bromide (MTT) assay and the fluorescence intensity was analyzed using an ArrayScan VTI HCS Reader (Thermo Scientific, Rockford, IL, USA). Subsequently, ESCs (day 0), HPCs (day 22 of differentiation) and HCs (day 40 of differentiation) were treated with four concentrations of DEN (0, 1, 5 and 15 mM; G1, G2, G3, G4, respectively) for 24 h.

### EpCAM and PCNA mRNA expression in ESC, HPCs and HCs

RNA was isolated from cultured cells using an easy-spin Total RNA Extraction kit (Intron Biotechnology, Scottsdale, AZ, USA), dissolved in DEPC-treated distilled water and stored at −80°C until use. RNA concentrations were measured using a UV/Vis Spectrophotometer (DU730; Beckman Coulter, Miami, FL, USA). The quality of the isolated RNA was assessed using an Agilent 2100 Bioanalyzer (Agilent, Palo Alto, CA, USA) and an Agilent RNA 6000 Nano kit (Agilent).

EpCAM and PCNA mRNA expression was determined by relative quantitative real-time PCR in 96-well optical plates using an ABI 7500 Real Time PCR System (Applied Biosystems, Foster City, CA, USA). Assay-on-Demand TaqMan probes (Applied Biosystems) were used to measure EpCAM and PCNA mRNA (Table I). A master mix was created containing the following: 6.25 *μ*l water, 1.25 *μ*l forward primer (9 *μ*M) and reverse primer (9 *μ*M), 2.5 *μ*l probe mixture (2.5 *μ*M) and 12.5 *μ*l TaqMan PCR 2X master mixture (Applied Biosystems). Reverse transcribed total RNA (40 ng in 5 *μ*l) was added as the PCR template.

The following PCR conditions were used: initial activation of uracyl-*N*-glycosylase at 50°C for 2 min; activation of AmpliTaq Gold at 95°C for 10 min; and 45 cycles of denaturation at 95°C for 15 sec and annealing/extension at 60°C for 1 min. During PCR, the amplified products were continuously monitored by measuring the fluorescence emission. All PCR assays were performed in triplicate.

The expression levels of the target genes were normalized to mouse GAPDH mRNA and were presented as relative expression. The expression of the genes was normalized to GAPDH, using the comparative C_t_ method. The cycle number at which the fluorescence signal of the target product was detectable (threshold cycle, C_t_) was normalized against the C_t_ of GAPDH, to give ΔC_t_. The expression of the genes relative to a reference was calculated as 2^−ΔΔCt^, where ΔΔC_t_ referred to the difference between the ΔCt values of the test group and the reference.

### Statistical analysis

Data were analyzed using the Student’s t*-*test with JMP software (SAS Institute, Cary, NC, USA). For all comparisons, P<0.05 was considered to indicate a statistically significant difference.

## Results

### Histopathological examination of hepatic masses

Histo-pathological examination of the 71 hepatic masses showed that 48 samples were adenomas and 23 were adenocarcinomas. The tumor tissues exhibited nuclear pleomorphism and alteration of cellular structure, with or without fatty liver and inflammatory cell infiltration.

### Immunohistochemical examination of EpCAM

**Figure 1:**
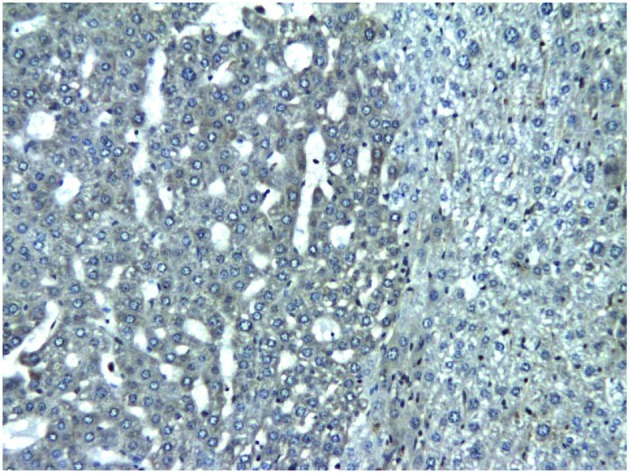
EpCAM expression in a DEN-induced hepatocellular tumor. Magnification ×200. Note the high expression levels of EpCAM and its cytoplasmic localization in the liver tissue of the DEN-induced tumor. EpCAM, epithelial cell adhesion molecule; DEN, diethylnitrosamine.

EpCAM was immunohistochemically detected mainly in hepatic tumor cells, with some expression in the surrounding visually normal cells, and exhibited a cytoplasmic staining pattern (Fig. 1). EpCAM expression was similar in benign and malignant tumors.

### Double staining of EpCAM and PCNA

**Figure 2:**
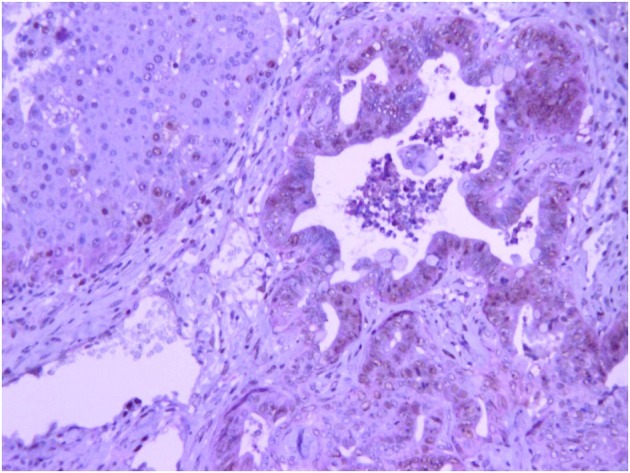
Co-expression of PCNA and EpCAM in a DEN-induced tumor. Magnification ×200. Note the nuclear staining of PCNA (brown) and the cytoplasmic staining of EpCAM (red) in the tumor and the co-expression in dysplastic ductal tumor cells. PCNA, proliferating cell nuclear antigen; EpCAM, epithelial cell adhesion molecule; DEN, diethylnitrosamine.

Double staining showed that EpCAM and PCNA were co-expressed in numerous tumor cells, particularly in dysplastic ductal tumor cells (Fig. 2). PCNA showed nuclear staining and EpCAM exhibited cytoplasmic staining.

### EpCAM and PCNA mRNA expression in ESCs, HPCs and HCs

**Figure 3:**
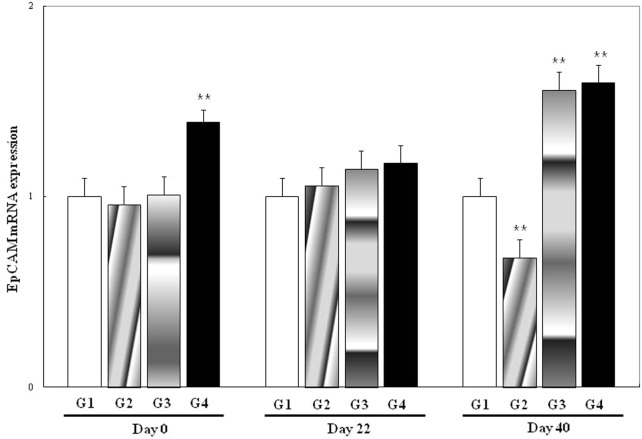
Expression of epithelial cell adhesion molecule (EpCAM) mRNA in mouse embryonic stem cells (ESCs), hepatic progenitor cells (HPCs) and hepatocyte-like cells (HCs). ESCs (day 0), HPCs (day 22) and HCs (day 40) were treated with DEN at four doses (0, 1, 5 and 15 mM; G1, G2, G3 and G4, respectively) for 24 h and the expression of EpCAM mRNA was determined by PCR. ^**^Significantly different from G1 (P<0.01). DEN, diethylnitrosamine.

The expression of EpCAM mRNA was significantly different for G4 at day 0 (P<0.01) and for G2, G3 and G4 at day 40 (P<0.01) compared with the control (G1) at the corresponding time-points (Fig. 3). There were no differences for G2 or G3 at day 0, or for G2, G3 or G4 at day 22.

**Figure 4:**
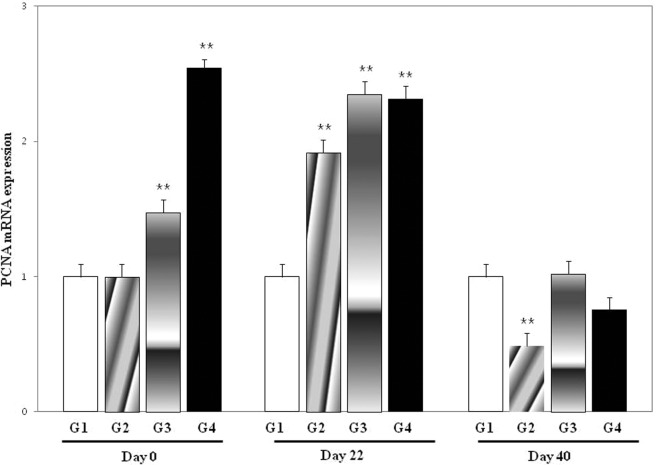
Expression of proliferating cell nuclear antigen (PCNA) mRNA in mouse embryonic stem cells (ESCs), hepatic progenitor cells (HPCs) and hepatocyte-like cells (HCs). ESCs (day 0), HPCs (day 22) and HCs (day 40) were treated with DEN at four doses (0, 1, 5 and 15 mM; G1, G2, G3 and G4, respectively) for 24 h and the expression of PCNA mRNA was determined by PCR. ^**^Significantly different from G1 (P<0.01). DEN, diethylnitrosamine.

PCNA mRNA expression was significantly different for G3 and G4 at day 0 (P<0.01), for G2, G3 and G4 at day 22 (P<0.01) and for G2 at day 40 (P<0.01) compared with G1 at the corresponding time-points (Fig. 4). There were no differences for G2 at day 0 and for G3 or G4 at day 40.

## Discussion

In the present study, EpCAM expression was increased in DEN-induced tumors and was associated with PCNA. Although EpCAM expression was also detected in surrounding visually normal cells, its expression was stronger in hepatic tumor cells. It has been reported that EpCAM-positive HCCs express stem cell and progenitor cell markers (9). Double staining revealed that EpCAM and PCNA were co-expressed in numerous tumor cells, suggesting that EpCAM-positive tumor cells may have the potential to proliferate.

Previously, EpCAM was identified as an additional marker of cancer-initiating cells (7) and it was detected in the cytoplasm of hepatic stem cells and in the plasma membranes of hepatoblasts (2). EpCAM-positive cells in the rat liver were shown to be bipotential adult hepatic epithelial progenitors (17). These findings indicate that EpCAM may be involved in the early stages of liver carcinogenesis.

To investigate the role of EpCAM in cancer initiation, we examined its expression in hepatic cells at various stages of differentiation. Hepatic differentiation of mouse ESCs may be induced in a stepwise manner by adding several specific growth factors following embryoid body formation (18) and functional hepatocytes may be generated using chemically defined culture conditions (19). In the present study, mouse NVRQS-11F ESCs were efficiently differentiated into hepatic lineage cells, HPCs and HCs. This multi-step generation of HCs from ESCs resembles *in vivo* hepatogenesis and ESC-derived hepatogenesis may be useful as a novel integrative model for hepatocarcinogenesis or for the hepatic toxicity evaluation of a number of chemicals.

DEN treatment enhanced EpCAM expression in ESCs, although EpCAM expression may be lost as the progeny of adult liver stem cells differentiate toward HPCs. However, DEN treatment induced the upregulation of EpCAM in HCs. This indicates that carcinogen treatment altered EpCAM expression in cells at each stage of differentiation.

Notably, DEN treatment increased PCNA expression at days 0 and 22, but not at day 40. This may be significant, since any proliferative cell in the liver may be susceptible to neoplastic transformation at the time of carcinogen exposure. As there was a time lag between increased EpCAM expression and cell proliferation, additional stem cell proteins may be involved. Further studies are also warranted to investigate the role of other stem cell markers in the context of hepatocarcinogenesis.

It has been hypothesized that stem cells and/or progenitor cells are transformed into cancer stem cells (20) by a process involving the dysregulation of stem cell self-proliferation (21). Considering that the induction of hepatic tumors by DEN treatment in animals was dependent on age at carcinogen treatment (22), there may be more hepatic stem and/or progenitor cells in young animals. Generally, compared with older animals, young animals are more sensitive to chemical-induced carcinogenesis (23). Hepatic tumors have been generated in young animals upon the administration of only a single injection of DEN (22) and ∼1% of cells treated with a tumor promoter developed into altered hepatic foci (24).

In the present study, low-dose DEN treatment decreased cell proliferation at day 40. Although it is not known why cell proliferation was not observed at day 40 following middle- and high-dose DEN treatment, it may be that the differentiation stage of hepatic cells is a significant factor in cellular proliferation caused by carcinogen treatment. Further studies are required to investigate the effects of various hepatocarcinogens on hepatic cells at various stages of differentiation.

In summary, EpCAM and PCNA expression was increased in DEN-induced tumors and the expression of EpCAM and PCNA in ESCs, HPCs and HCs was modulated by DEN treatment. This study contributes to cancer research by clarifying EpCAM expression in hepatic tumors and during the early stages of hepatocarcinogenesis.

## Figures and Tables

**Table I:** Probe sequences.

Assay ID	Probe sequence	Gene symbol	Amplicon size (bp)
Mm00493214_m1	TTGAAAAAGATGTGAAGGGGGAGTC	EpCAM	95
Mm00448100_g1	CAACTTGGAATCCCAGAACAGGAGT	PCNA	117
